# Simple, Low-Cost Detection of *Candida parapsilosis* Complex Isolates and Molecular Fingerprinting of *Candida orthopsilosis* Strains in Kuwait by ITS Region Sequencing and Amplified Fragment Length Polymorphism Analysis

**DOI:** 10.1371/journal.pone.0142880

**Published:** 2015-11-18

**Authors:** Mohammad Asadzadeh, Suhail Ahmad, Ferry Hagen, Jacques F. Meis, Noura Al-Sweih, Ziauddin Khan

**Affiliations:** 1 Department of Microbiology, Faculty of Medicine, Kuwait University, Safat, Kuwait; 2 Department of Medical Microbiology and Infectious Diseases, Canisius Wilhelmina Hospital, Nijmegen, The Netherlands; 3 Department of Medical Microbiology, Radboudumc, Nijmegen, The Netherlands; California Department of Public Health, UNITED STATES

## Abstract

*Candida parapsilosis* has now emerged as the second or third most important cause of healthcare-associated *Candida* infections. Molecular studies have shown that phenotypically identified *C*. *parapsilosis* isolates represent a complex of three species, namely, *C*. *parapsilosis*, *C*. *orthopsilosis* and *C*. *metapsilosis*. *Lodderomyces elongisporus* is another species phenotypically closely related to the *C*. *parapsilosis*-complex. The aim of this study was to develop a simple, low cost multiplex (m) PCR assay for species-specific identification of *C*. *parapsilosis* complex isolates and to study genetic relatedness of *C*. *orthopsilosis* isolates in Kuwait. Species-specific amplicons from *C*. *parapsilosis* (171 bp), *C*. *orthopsilosis* (109 bp), *C*. *metapsilosis* (217 bp) and *L*. *elongisporus* (258 bp) were obtained in mPCR. Clinical isolates identified as *C*. *parapsilosis* (n = 380) by Vitek2 in Kuwait and an international collection of 27 *C*. *parapsilosis* complex and *L*. *elongisporus* isolates previously characterized by rDNA sequencing were analyzed to evaluate mPCR. Species-specific PCR and DNA sequencing of internal transcribed spacer (ITS) region of rDNA were performed to validate the results of mPCR. Fingerprinting of 19 clinical *C*. *orthopsilosis* isolates (including 4 isolates from a previous study) was performed by amplified fragment length polymorphism (AFLP) analysis. Phenotypically identified *C*. *parapsilosis* isolates (n = 380) were identified as *C*. *parapsilosis* sensu stricto (n = 361), *C*. *orthopsilosis* (n = 15), *C*. *metapsilosis* (n = 1) and *L*. *elongisporus* (n = 3) by mPCR. The mPCR also accurately detected all epidemiologically unrelated *C*. *parapsilosis* complex and *L*. *elongisporus* isolates. The 19 *C*. *orthopsilosis* isolates obtained from 16 patients were divided into 3 haplotypes based on ITS region sequence data. Seven distinct genotypes were identified among the 19 *C*. *orthopsilosis* isolates by AFLP including a dominant genotype (AFLP1) comprising 11 isolates recovered from 10 patients. A rapid, low-cost mPCR assay for detection and differentiation of *C*. *parapsilosis*, *C*. *orthopsilosis*, *C*. *metapsilosis* and *L*. *elongisporus* has been developed.

## Introduction

The incidence of candidemia is increasing worldwide particularly among hospitalized patients [1]. *Candida parapsilosis* is now recognized as the second or third most common *Candida* spp. associated with invasive candidiasis [1,2]. Three distinct groups were recognized among clinical *C*. *parapsilosis* isolates and group II and group III isolates were subsequently raised to two new species named; *Candida orthopsilosis* and *Candida metapsilosis*, respectively [3]. Furthermore, *Lodderomyces elongisporus* is another species phenotypically closely related to *C*. *parapsilosis* complex [4]. Infections caused by *C*. *parapsilosis* sensu stricto are more frequent than those caused by other *C*. *parapsilosis*-complex members [5–10]. However, species-specific identification of *C*. *parapsilosis* complex is important in patient management and choosing the appropriate treatment due to differences in antifungal susceptibly profile [5,9,11,12]. Phenotypically, it is not possible to distinguish these closely related species. Identification of *C*. *orthopsilosis*, *C*. *metapsilosis* and *L*. *elongisporus* from *C*. *parapsilosis* is usually accomplished by molecular techniques [3–5]. Many molecular methods have been developed for this purpose and these typically involve PCR amplification followed by restriction fragment length polymorphism (PCR-RFLP) or DNA sequencing of different genes [3,13–17]. Matrix-assisted laser desorption/ionization time-off-light mass spectrometry (MALDI-TOF-MS) analyses [18,19] and real-time PCR (qPCR) assays involving probe primers [20] or high resolution melting curve (HRM) analysis [21,22] have also been described. However, these techniques are laborious, time-consuming and technically demanding or require expensive reagents/equipment.

Fingerprinting studies are performed to ascertain the source of infection. Although fingerprinting studies of *C*. *parapsilosis* sensu stricto isolates have been extensively carried out, only few studies have explored the genotypic heterogeneity among clinical *C*. *orthopsilosis* and *C*. *metapsilosis* isolates. Unlike other *Candida* spp. (e.g. *C*. *albicans*, *C*. *tropicalis*, *C*. *parapsilosis* etc.), highly discriminatory and portable multilocus sequence typing and/or microsatellite typing schemes have not yet been developed for *C*. *orthopsilosis*. Amplified fragment length polymorphism (AFLP) analysis was first used as a reliable method for identification and strain typing of *C*. *parapsilosis* complex isolates [23] and was recently shown to be more discriminatory for typing of *C*. *orthopsilosis* in comparison with MALDI-TOF-MS [18]. AFLP analyses have also shown persistence of strains in the hospital environments for years forming reservoirs of infection as well as during infection of patients with *C*. *orthopsilosis* [6,23]. However, only few studies have been performed on *C*. *orthopsilosis* strains and the population structure of this species remains unknown for many countries/regions.

In this study, we developed a simple, low-cost multiplex PCR (mPCR) assay for rapid detection and differentiation of the three closely related species (*C*. *parapsilosis*, *C*. *orthopsilosis* and *C*. *metapsilosis*) comprising *C*. *parapsilosis* complex as well as the phenotypically closely related species *L*. *elongisporus* in a single PCR assay. The method was evaluated by using 380 phenotypically identified *C*. *parapsilosis* strains isolated from clinical specimens in Kuwait and an international collection of 27 *C*. *parapsilosis* complex and *L*. *elongisporus* isolates previously characterized by rDNA sequencing. Furthermore, AFLP was performed for molecular fingerprinting of *C*. *orthopsilosis* isolates to determine their genetic relatedness.

## Materials and Methods

### Reference strains and clinical isolates

Reference strains of *C*. *parapsilosis* (ATCC 22019), *C*. *orthopsilosis* (ATCC 96139), *C*. *metapsilosis* (ATCC 96143), *L*. *elongisporus* (CBS 2605), *Candida albicans* (ATCC 90028), *Candida dubliniensis* (CD36), *Candida tropicalis* (ATCC 750), *Candida glabrata* (ATCC 15545), *Candida nivariensis* (CBS 9983), *Candida bracarensis* (CBS 10154), *Candida krusei* (ATCC 6258), and *Candida haemulonii* (CBS 5149) were used as reference *Candida* species. The clinical specimens including blood were collected from patients at various hospitals across Kuwait as part of routine patient care for the isolation of fungal pathogens. The clinical specimens were collected after obtaining verbal consent from patients as part of routine diagnostic work-up. The isolates were sent to Mycology Reference Laboratory, Department of Microbiology, Faculty of Medicine, Kuwait University for identification and antifungal susceptibility testing. A total of 380 clinical isolates of *Candida parapsilosis*-complex and identified by Vitek2 yeast identification system (bioMérieux, Marcy-l’Etoile, France) were selected from the culture collection maintained in the department and were analyzed in this study. These isolates originated from blood (n = 221), sputum and other respiratory specimens (n = 45), urine (n = 37) and various other (ear/eye/skin/wound swabs, nail scrapings, catheter tips and cerebrospinal fluid) specimens (n = 77). In addition, 27 epidemiologically unrelated yeast isolates characterized by ITS region sequencing were also tested to ascertain the robustness of the mPCR assay. These included *C*. *parapsilosis* sensu stricto (n = 12), *C*. *orthopsilosis* (n = 9), *C*. *metapsilosis* (n = 5) and *L*. *elongisporus* (n = 1) strains isolated from blood and other clinical specimens in Netherlands, Brazil, Saudi Arabia and Germany. The study was approved by the Joint Committee for the Protection of Human Subjects in Research, Health Sciences Center, Kuwait University and Ministry of Health, Kuwait.

### Phenotypic identification

All 380 *C*. *parapsilosis* isolates, previously identified by Vitek2 were grown on CHROMagar Candida (Becton Dickinson, Bootle, UK) and the results were interpreted according to manufacturer’s instructions. The typical pink/lavender color of *C*. *parapsilosis* [4, 24–26] was used for identifying these isolates as belonging to *C*. *parapsilosis*-complex.

### Template DNA preparation and multiplex PCR assay

The genomic DNA from reference strains and clinical isolates of different *Candida* species was extracted by using Gentra Puregene Yeast DNA extraction kit (Qiagen, Hilden Germany) according to kit instructions or by the rapid method using Chelex-100. A loop full of yeast colony grown on Sabouraud dextrose agar plate was suspended in 1 ml of sterile water in a microcentrifuge tube containing 50 mg Chelex-100 (Sigma-Aldrich Co., St. Louis, MO, USA), the contents were heated at 95°C for 20 min and then centrifuged. The supernatant was transferred to a new tube and typically 2 μl was used for mPCR.

Four different forward primers targeting specific sequences within ITS-1 region of rDNA of the four species and one common reverse primer targeting 5.8S rRNA gene, were synthesized for PCR amplification (Table 1). The primer sequences were selected based on multiple sequence alignment of ITS region sequences of multiple strains of all commonly encountered clinical yeast species that are available from the GenBank. The species specificity of the primers mCPF, mCOF, mCMF, and mLEF for *C*. *parapsilosis*, *C*. *orthopsilosis*, *C*. *metapsilosis* and *L*. *elongisporus* respectively, and a common reverse primer (mCPCR) for these four species, was further tested by performing BLAST searches (http://www.ncbi.nlm.nih.gov/entrez/query.fcgi). All four species-specific primers showed complete identity with the available sequences deposited in the data bank for the corresponding species that have previously been isolated and sequenced at different geographic locations around the world. PCR amplification was performed in a final volume of 50 μl containing 1x AmpliTaq DNA polymerase buffer I and 1 unit of AmpliTaq DNA polymerase (Applied Biosystems, Brachburg, NJ, USA), 10 pmol of mCPF, mCOF, mCMF, mLELF and mCPCR primers, 2 μl of template DNA and 100 μM of each dNTP. Cycling conditions included an initial denaturation at 95°C for 5 min followed by 30 cycles of 95°C for 1 min, 52°C for 30 s and 72°C for 1 min and a final extension at 72°C for 10 min. PCR products (20 μl) were run on 2% (w/v) agarose gels, as described previously [27].

**Table 1:** Specific features and DNA sequences of the mPCR primers used in this study.

No.	Primer name	Target region	Direction	Species specificity	DNA Sequence	Amplicon size (bp)^a^
1	mCPF	ITS-1	Forward	*C*. *parapsilosis*	5’-TTTGCTTTGGTAGGCCTTCTA-3’	171
2	mCOF	ITS-1	Forward	*C*. *orthopsilosis*	5’-TAAGTCAACTGATTAACTAAT-3’	109
3	mCMF	ITS-1	Forward	*C*. *metapsilosis*	5’-AACTGCAATCCTTTTCTTTCTA-3’	217
4	mLEF	ITS-1	Forward	*L*. *elongisporus*	5’-TACAGAATTTTGAGAATTGTG-3’	258
5	mCPCR	5.8S rRNA	Reverse	*C*. *parapsilosis*-complex	5’-AATATCTGCAATTCATATTACT-3’	-

^a^The amplicon size is based on the combination of the four individual forward primers together with the reverse primer

### Species-specific amplification of ITS region of rDNA

The results of mPCR were confirmed by using species-specific PCR amplification for all *C*. *orthopsilosis*, *C*. *metapsilosis* and *L*. *elongisporus* and 50 randomly selected *C*. *parapsilosis* sensu stricto isolates. The primer sequences for species-specific amplification of rDNA from *C*. *parapsilosis* sensu stricto, *C*. *orthopsilosis* and *C*. *metapsilosis* were the same as described previously [13] while primers LELF (5’-TGGCTGCTTAATTGCTCTGCT-3’) and LELR (5’-TAAGCACAATGGAGTGGTTAG-3’) were used for species-specific amplification of DNA (expected size of 355 bp amplicon) from *L*. *elongisporus*. Other reaction and cycling conditions during PCR amplification were same as described above and detection of amplicons was performed by agarose gel electrophoresis as described previously [13,27].

### DNA sequencing of the ITS region and the D1/D2 domains of 28S rDNA gene

The results of species-specific identification of all *C*. *orthopsilosis*, *C*. *metapsilosis* and *L*. *elongisporus* isolates and 10 randomly selected *C*. *parapsilosis* sensu stricto isolates were also confirmed by DNA sequencing of the ITS region and/or the D1/D2 domains of 28S rDNA and both strands were sequenced. PCR amplification of ITS region and D1/D2 domains of 28S rDNA and sequencing reactions were performed as described previously [28,29]. BLAST searches (http://blast.ncbi.nlm.nih.gov/Blast.cgi?) were performed and >99% sequence identity was used for species identification. Pairwise comparisons and multiple sequence alignments were also performed with CLUSTAL W2. Phylogenetic tree was constructed with MEGA5.2 software using the neighbor-joining method with the pair-wise deletion of gaps option and the maximum composite likelihood model. The robustness of the branches was assessed by bootstrap analysis with 1000 replicates.

### Fingerprinting of *C*. *orthopsilosis* isolates by AFLP analysis

All *C*. *orthopsilosis* isolates (n = 15) identified in this study and 4 well-characterized *C*. *orthopsilosis* isolates available from our previous study [13] were typed by AFLP fingerprint analysis. These isolates were collected over a 17-year period (1997 to 2013). Of the 19 isolates, 13 were recovered from different anatomic sites of 13 individual patients while repeat isolates (2 isolates each) were obtained from 3 patients. Repeat isolates were used to ascertain the reproducibility of the AFLP data and to see whether *C*. *orthopsilosis* isolates recovered from different anatomic sites of the same patient are genotypically identical or different. For this purpose, genomic DNA was extracted by using the Roche MagNA Pure 96 platform which yields a priori highly pure nucleic acids from pure cultures as described previously [30]. The AFLP analysis was carried out by using 50 ng of genomic DNA was mixed with restriction ligation containing EcoR1 and MseI restriction enzymes (New England Biolabs, Beverly, MA, USA) and complementary adaptors as described previously [30]. Prior to further use, the restriction–ligation reaction was diluted by adding 80 μl of 10 mM Tris-HCl (pH 8.3) buffer. One microliter of the diluted product was used for amplification in a final volume of 25 μl by using the selective primers EcoR1 (5′-FLU-GACTGCGTACCAATTCAC-3′) and MseI (5′-GATGAGTCCTGACTAAC-3′) [30]. One microliter of a 10-fold dilution of amplified products was added to a mixture of 8.9 μl of water and 0.1 μl of LIZ600 internal size marker (Applied Biosystems), followed by fragment analysis on an ABI 3500xL Genetic Analyzer according to the instructions of the manufacturer (Applied Biosystems). *Candida orthopsilosis* reference strain (ATCC 96139) was used as a control and a clinical *C*. *metapsilosis* isolate (Kw164-7/12) was used as an out group. Raw data were analyzed by using Bionumerics v6.6 software (Applied Maths, Sint-Martens-Latem, Belgium) and a dendrogram was generated using standard Pearson and unweighted pair group method with arithmetic mean (UPGMA) settings.

### Antifungal drug susceptibility testing

*In vitro* activity of amphotericin B (AP), fluconazole (FL), 5-flucytosine (FC), voriconazole (VO) and caspofungin (CS) was determined by the Etest (AB BIODISK, Solna, Sweden) in accordance with the manufacturer’s instructions and as described in detail previously [31]. The minimum inhibitory concentration (MIC) values were recorded after 48 h of incubation at 35°C. The interpretive susceptibility breakpoints as recommended by Clinical Laboratory Standards Institute (CLSI) were used for fluconazole, flucytosine and voriconazole. Due to lack of defined breakpoints for amphotericin B, isolates having an MIC < 2.0 mg/L were considered as susceptible. In case of caspofungin an isolate with MIC ≤ 2 mg/L was recorded as susceptible [32]. Quality control was ensured by testing *C*. *parapsilosis* ATCC 22019 and *C*. *albicans* ATCC 90028, as recommended by CLSI.

## Results

### Phenotypic identification of *C*. *parapsilosis-*complex isolates

A total of 380 clinical yeast isolates identified as belonging to *C*. *parapsilosis*-complex on the basis of conventional biochemical tests were screened to identify *C*. *parapsilosis* sensu stricto, *C*. *orthopsilosis*, *C*. *metapsilosis* and *L*. *elongisporus* isolates. Based on CHROMagar Candida, 3 isolates produced turquoise blue color and were tentatively identified as *L*. *elongisporus*—while the remaining 377 isolates produced pink/lavender colonies and were identified as *C*. *parapsilosis*-complex members (Table 2, S1 Fig).

**Table 2:** Comparison between various phenotypic and genotypic methods used for identification of *C*. *parapsilosis* complex isolates.

Method	No. (%) of isolates identified as			
	*C*. *parapsilosis*	*C*. *orthopsilosis*	*C*. *metapsilosis*	*L*. *elongisporus*
Vitek2 identification system	380	-	-	-
CHROMagar Candida	377*	-	-	3**
New multiplex-PCR	361	15	1	3

*All 377 *C*. *parapsilosis* isolates showed pink/lavender color typical for *C*. *parapsilosis*-complex isolates.

**All 3 *L*. *elongisporus* isolates showed turquoise color atypical for *C*. *parapsilosis*-complex isolates.

### Genotypic identification of *C*. *parapsilosis* complex isolates by mPCR

**Fig 1:**
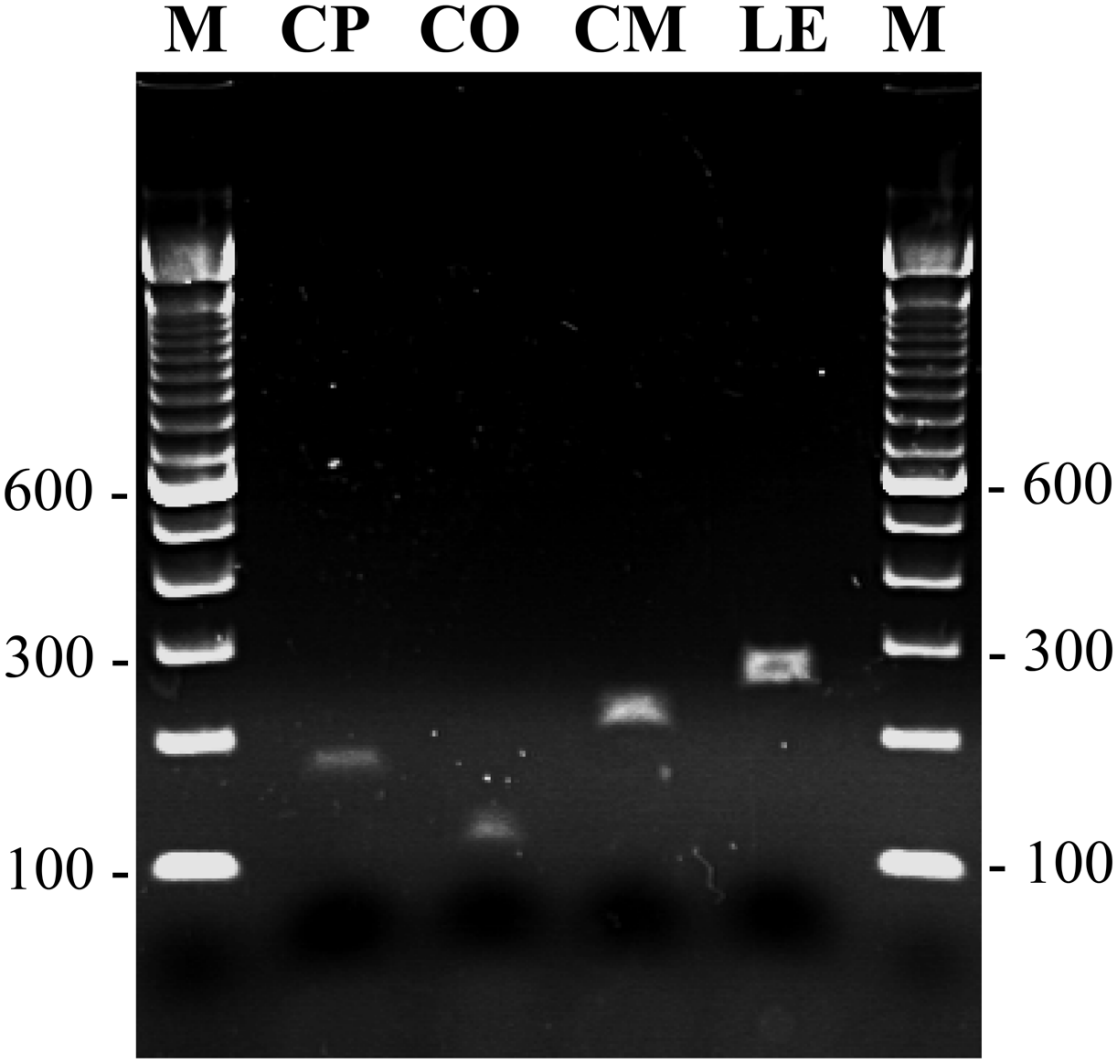
Detection of mPCR amplified products by agarose gel electrophoresis. An agarose gel of mPCR amplified products using template DNA from reference strain of *C*. *parapsilosis* (lane CP), *C*. *orthopsilosis* (lane CO), *C*. *metapsilosis* (lane CM) and *L*. *elongisporus* (lane LE). Lane M is 100 bp DNA marker and the position of migration of 100 bp, 300 bp and 600 bp fragments are marked.

mPCR amplification performed with mCPF + mCOF + mCMF + mLEF + mCPCR primers yielded an expected size amplicon of nearly 171 bp, 109 bp, 217 bp and 258 bp with DNA extracted by both, the rapid Chelex-100 method as well as by the commercial kit from the reference strains of *C*. *parapsilosis*, *C*. *orthopsilosis*, *C*. *metapsilosis* and *L*. *elongisporus*, respectively (Fig 1). No amplicon was obtained with genomic DNA prepared from reference strains of *C*. *albicans*, *C*. *dubliniensis*, *C*. *tropicalis*, *C*. *glabrata*, *C*. *nivariensis*, *C*. *bracarensis*, *C*. *krusei* and *C*. *haemulonii*, as expected. mPCR amplification of DNA from 380 clinical *C*. *parapsilosis* isolates speciated by Vitek2 yeast identification system identified 361 isolates as *C*. *parapsilosis* sensu stricto, 15 isolates as *C*. *orthopsilosis*, 1 isolate as *C*. *metapsilosis* and 3 isolates as *L*. *elongisporus* (Table 2). Again, genomic DNA extracted by both methods yielded identical results. The mPCR results were confirmed for all 15 *C*. *orthopsilosis*, 1 *C*. *metapsilosis*, 3 *L*. *elongisporus* and 50 randomly selected *C*. *parapsilosis* sensu stricto isolates by employing species-specific PCR amplification and/or by direct DNA sequencing of ITS region and D1/D2 domains of rDNA. The DNA sequence data in each case confirmed the results of species-specific identification by mPCR. The DNA sequences have been submitted to European Molecular Biology Laboratory (EMBL) databank under accession no. LN864540-LN864563. The results were further validated by testing an international collection of 27 yeast isolates characterized by ITS region sequencing which included *C*. *parapsilosis* sensu stricto (n = 12), *C*. *orthopsilosis* (n = 9), *C*. *metapsilosis* (n = 5) and *L*. *elongisporus* (n = 1) strains as the mPCR assay detected each of the above isolate accurately.

### Molecular fingerprinting of *C*. *orthopsilosis* isolates

A total of 19 *C*. *orthopsilosis* isolates were available for fingerprinting studies. These isolates were collected from 16 patients over a period of 17 years. Repeat isolates were collected from 3 patients and were isolated from different clinical specimens (patients P4 and P15) or from the same specimen collected at different time intervals (patient P12) (Table 3). Based on ITS region sequence data, 3 distinct haplotypes (labeled as ITSA, ITSB and ITSC) were identified among 19 *C*. *orthopsilosis* isolates (Table 3).). Ten isolates were obtained from 8 neonates, while the remaining 9 isolates were from 8 adult patients. The length (516 nucleotides) and ITS region sequence of 5 haplotype ITSA isolates matched completely with the sequence from reference *C*. *orthopsilosis* strain ATCC 96139. Eleven *C*. *orthopsilosis* isolates belonged to haplotype ITSB that differed from haplotype ITSA in length (519 nucleotides in ITSB due to insertion of a ‘T’ residue at nucleotide positions 58, 144 and 414) and nucleotide substitutions at 2 other positions (S1 Table). Only 3 *C*. *orthopsilosis* isolates belonged to haplotype ITSC that also differed from haplotype ITSA in length (515 nucleotides in ITSC due to deletion of ‘T’ residues at nucleotide positions 78 and 79 and insertion of a ‘T’ residue at nucleotide position 144) and nucleotide substitutions at 2 other positions (S1 Table). The repeat isolates from the same patient exhibited identical ITS region sequence-based haplotype (Table 3).

**Table 3:** Source of isolation, ITS region sequence-based haplotypes, AFLP-based genotypes and antifungal drug susceptibility testing results for 19 clinical *Candida orthopsilosis* isolates used in this study.

Patient no.	Isolate no.	Source of isolation	ITS region (bp)	ITS-based haplotype	AFLP genotype	Minimum inhibitory concentration (MIC, mg/L) of				
						AP	FL	FC	VO	CS
P1	Kw301/97	Blood	516	ITSA	AFLP2	0.19	0.19	0.023	0.008	0.25
P2	Kw304/97	Blood	516	ITSA	AFLP3	0.125	0.75	0.032	0.047	0.25
P3	Kw1056/04	Blood	516	ITSA	AFLP7	0.5	1.5	0.094	0.094	0.38
**P4**	**Kw469/10**	**Rectal swab**	**516**	**ITSA**	**AFLP6**	**0.016**	**0.19**	**0.023**	**0.006**	**0.38**
**P4**	**Kw470/10**	**Urine**	**516**	**ITSA**	**AFLP6**	**0.012**	**0.38**	**0.023**	**0.032**	**0.38**
P5	Kw1782/06	Ear swab	519	ITSB	AFLP1	0.125	0.75	0.094	0.006	0.25
P6	Kw3372/07	Sputum	519	ITSB	AFLP1	0.023	0.38	0.023	0.094	0.25
P7	Kw1690/07	Rectal swab	519	ITSB	AFLP1	0.048	0.38	0.016	0.008	0.38
P8	Kw108/08	Sputum	519	ITSB	AFLP1	0.19	0.38	0.023	0.094	0.25
P9	Kw2238/09	Blood	519	ITSB	AFLP1	0.008	0.19	0.016	0.006	0.25
P10	Kw1078/10	Blood	519	ITSB	AFLP1	0.016	0.38	0.016	0.023	0.25
P11	Kw2949/11	Blood	519	ITSB	AFLP1	0.023	0.125	0.023	0.004	0.38
**P12**	**Kw674/11**	**Tracheal aspirate**	**519**	**ITSB**	**AFLP1**	**0.016**	**0.38**	**0.032**	**0.094**	**0.25**
**P12**	**Kw747/11**	**Tracheal aspirate**	**519**	**ITSB**	**AFLP1**	**0.125**	**0.75**	**0.016**	**0.064**	**0.25**
P13	Kw228-8/12	Blood	519	ITSB	AFLP1	0.047	0.25	0.023	0.016	0.25
P14	Kw313-12/13	Throat swab	519	ITSB	AFLP1	0.023	0.19	0.016	0.023	0.5
**P15**	**Kw105/10/13**	**Umbilical Tip**	**515**	**ITSC**	**AFLP4**	**0.008**	**0.25**	**0.032**	**0.47**	**0.25**
**P15**	**Kw106/10/13**	**Urine**	**515**	**ITSC**	**AFLP4**	**0.008**	**0.25**	**0.023**	**0.047**	**0.38**
P16	Kw96-11/12	Blood	515	ITSC	AFLP5	0.125	12	>32	0.125	4

Repeat isolates from the same patient are shown in bold.

AP, amphotericin B; FL, fluconazole; FC, 5-flucytosine; VO, voriconazole; CS, caspofungin

**Fig 2:**
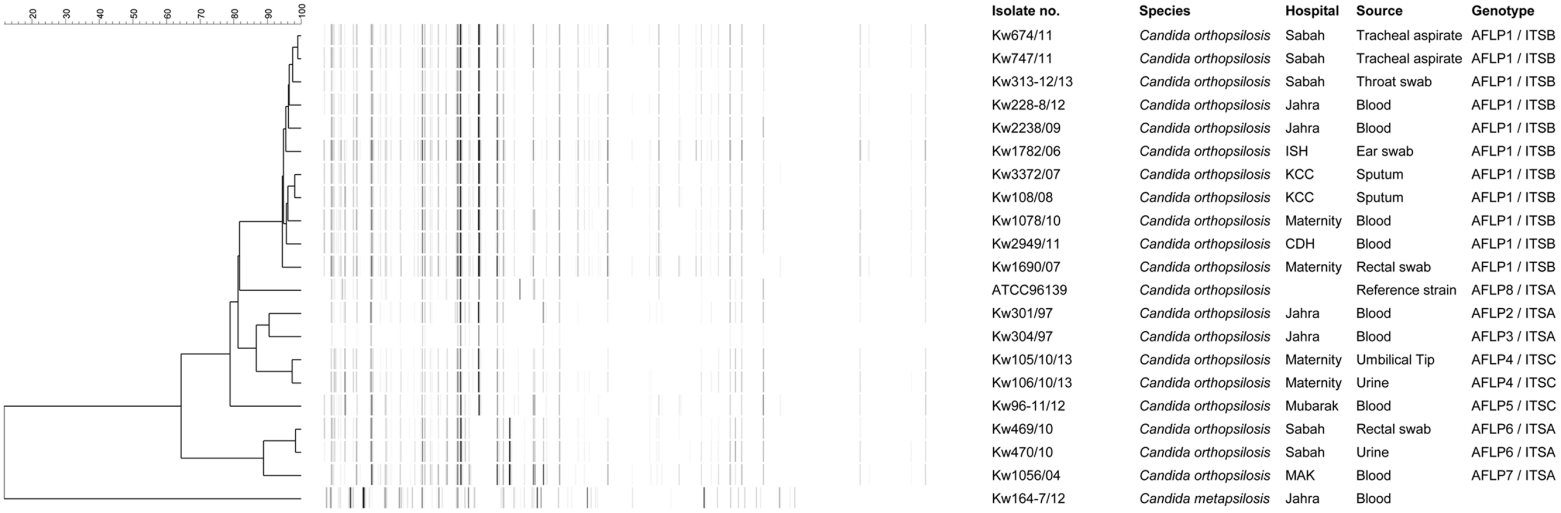
Amplified fragment length polymorphism (AFLP)-based fingerprinting of *C*. *orthopsilosis* isolates. An UPGMA-derived dendrogram based on AFLP fingerprints for 19 clinical *C*. *orthopsilosis* isolates obtained from 16 patients in Kuwait. The reference *C*. *orthopsilosis* strain (ATCC 96139) was also included in AFLP analysis and a clinical *C*. *metapsilosis* isolate (Kw164-7/12) was used as an outer group. Similarity is presented in percentages using the scale bar in the upper left corner. The columns after the AFLP patterns represent the isolate number, species name, hospital name, source of the isolates and AFLP and ITS genotypic grouping.

Compared to ITS region sequence-based data, the fingerprinting performed by AFLP analysis yielded greater genotypic heterogeneity among the 19 *C*. *orthopsilosis* isolates. A total of 102 fragments were obtained from *C*. *orthopsilosis* strains during AFLP analyses of which 32 (31.4%) were polymorphic fragments. An arbitrary cut-off value of ≤95% similarity among AFLP patterns was used for defining a distinct genotype and 7 genotypes (defined as AFLP1 to AFLP7) were identified among the 19 isolates (Fig 2 and Table 3). Repeat isolates from the same patient yielded the same genotype irrespective of whether they were isolated from the same specimen type or from clinical specimens originating from different anatomic sites (Table 3). Interestingly all 4 individual patient isolates belonging to ITS region sequence-based haplotype ITSA belonged to 4 (AFLP2, AFLP3, AFLP6 and AFLP7) different and unique AFLP genotypes, the reference *C*. *orthopsilosis* strain (ATCC 96139) also had a distinct genotype (ITSA, genotype AFLP8). Similarly, both individual patient isolates belonging to ITS region sequence-based haplotype ITSC belonged to 2 (AFLP4 and AFLP5) different and unique AFLP genotypes. On the contrary all 10 individual patient isolates belonging to ITS region sequence-based haplotype ITSB exhibited the same AFLP fingerprinting pattern (AFLP1) (Fig 2 and Table 3).

### Antifungal drug susceptibility testing data

All *C*. *orthopsilosis* isolates were susceptible to amphotericin B, fluconazole, 5-flucytosine, voriconazole and caspofungin (Table 3). Only one isolate, Kw96-11/12, showed reduced susceptibility/resistance to fluconazole (MIC = 12 mg/L), flucytosine (MIC = >32 mg/L) and caspofungin (MIC = 4 mg/L) by Etest.

## Discussion

*Candida orthopsilosis* and *C*. *metapsilosis* are two recently described species that are typically misidentified as *C*. *parapsilosis* by culture-based phenotypic methods. These species exhibit differences in minimum inhibitory concentrations (MICs) for antifungal drugs warranting species-specific identification [5,9,11,12]. Furthermore, it has been shown that the species identified from the catheter is not always responsible for the bloodstream infection [6]. Application of molecular methods have led to the identification of these two species in archived as well as in recently isolated *C*. *parapsilosis* strains [3,5,7,11–13,33]. These studies have shown that *C*. *orthopsilosis* and *C*. *metapsilosis* have worldwide distribution; however, the percent occurrence of these two individual species among *C*. *parapsilosis* complex isolates at various geographical locations varies considerably [3,5,6,8,13,33]. Furthermore, the occurrence of these species in various clinical specimens in several countries/geographical locations remain unknown due to the requirement of technically demanding and complex molecular methods which are still not routinely available in many developing countries.

In this study, we have developed a simple, low-cost mPCR assay for accurate identification of *C*. *parapsilosis*-complex (*C*. *parapsilosis*, *C*. *orthopsilosis*, *C*. *metapsilosis*) isolates as well as *L*. *elongisporus*, another closely related species in a single PCR assay. The robustness of the mPCR assay was evident from the analysis of 380 clinical isolates of *Candida parapsilosis*-complex in Kuwait and an international collection of 12 *C*. *parapsilosis* sensu stricto, 9 *C*. *orthopsilosis*, 5 *C*. *metapsilosis* and one *L*. *elongisporus* strain. The mPCR assay can be completed within 4 hours using basic PCR and gel electrophoresis equipment that are readily available in most clinical microbiology laboratories and will cost nearly 1–1.5 US$ per sample (depending on whether DNA is extracted by the Chelex-100 method or by the commercial kit and excluding the cost of culture and personnel time). Previously, species-specific identification has been achieved by PCR-RFLP assays that require time-consuming additional step of restriction enzyme digestion [3,13,17,34], by PCR sequencing of rDNA that is expensive and technically demanding [3,13,15] or by real-time PCR assays requiring expensive reagents and/or equipment [17,20–22]. Although rapid (<30 min) identification of *C*. *orthopsilosis* and *C*. *metapsilosis* is possible with MALDI-TOF MS analysis, the requirement for fresh cultures often necessitates sub-culturing for obtaining interpretable results and these two species are included in the database of only the Bruker Daltonics Biotyper system but not in the database of the Biomerieux Vitek MS system [18,19,35,36]. Another advantage of our mPCR assay is the simultaneous detection of *L*. *elongisporus* with no additional steps or cost.

The occurrence of 15 *C*. *orthopsilosis* isolates (4%) among 380 *C*. *parapsilosis* sensu lato strains is nearly the same as that reported in a previous study (4.4%) from Kuwait [13] and is within the global range of 1%–28% described in various studies [3,5,6,8,33]. Consistent with earlier reports, 14 of 15 *C*. *orthopsilosis* isolates were also susceptible to all antifungal drugs tested while only one isolate showed reduced susceptibility or resistance to some antifungal drugs [5,9,11,12]. Although *C*. *orthopsilosis* strains are typically isolated from adult patients [37], however, 10 of 19 of our *C*. *orthopsilosis* isolates originated from 8 neonates. Only few previous studies have reported the isolation of this species from blood cultures of neonates borne prematurely and young children [38–40].

Only few studies have been carried out to study the population structure and genetic diversity among clinical *C*. *orthopsilosis* isolates. While DNA sequencing of ITS region of rDNA identifies major haplotypes, more extensive fingerprinting studies have been carried out by using AFLP analyses [18,23,41]. We studied the population structure of 19 *C*. *orthopsilosis* isolates by ITS region sequence comparisons and AFLP analyses. No other previous study has performed fingerprinting of *C*. *orthopsilosis* strains by these two techniques simultaneously. Based on DNA sequence data of ITS region, 3 haplotypes (ITSA, ITSB and ITSC) were identified among 19 *C*. *orthopsilosis* isolates obtained from 16 patients with ITSA sequence showing complete identity with the corresponding sequence from *C*. *orthopsilosis* type strain (ATCC 96139). Only two (ITSA and ITSB) haplotypes were identified in our previous study from Kuwait [13]. Repeat isolates cultured from the same or different clinical specimens from the same patient were found to belong to the same ITS haplotype and no specific association was found between the source of the clinical sample, drug resistance profile and gender or nationality of the patient with a particular ITS haplotype. Similar to our study, three haplotypes were also identified by ITS region sequence comparisons among *C*. *orthopsilosis* isolates in a recent study from Brazil with ITSA, ITSB and ITSC corresponding to Brazilian haplotype 1, haplotype 3 and haplotype 2, respectively [41]. Previous studies have also shown the occurrence of three ITS haplotypes among *C*. *orthopsilosis* isolates from some geographical locations. Tay et al. [42] also detected three ITS haplotypes (labeled as P2, P3 and P3 variant) among *C*. *orthopsilosis* isolates from Malaysia. However, the ITS region sequence of only P2 grouping is identical to our ITSA isolates while there are differences between P3 isolates and the P3 variant isolate with our ITSB or ITSC isolates. Sai et al. [43] sequenced the ITS region from 13 *C*. *orthopsilosis* isolates and also identified 3 haplotypes even though they recognized only two main types (Type 1 and Type 2) as the lone intermediate isolate (C_ort_T2_90_125) was clubbed together with other Type 2 isolates. The placement of the C_ort_T2_90_125 isolate with other Type 2 isolates was supported by the analysis of the mating type locus. The ITS region sequence of our ITSA and ITSB haplotypes matched completely with the sequence of Type 1 and Type 2 isolates, respectively. However, the sequence of our ITSC haplotype did not match with the intermediate isolate (C_ort_T2_90_125) described by Sai et al. [43]. These studies have also suggested greater genotypic heterogeneity among clinical *C*. *orthopsilosis* isolates compared to *C*. *parapsilosis* sensu stricto isolates which are predominantly clonal and exhibit limited genotypic variations [13,41–44].

As expected, greater genotypic heterogeneity was detected among the isolates by AFLP analyses. A cut-off value of ≤95% similarity index among AFLP patterns was used for defining a distinct genotype. Repeat isolates from the same patient exhibiting ≥98% similarity index validated the cut-off value of ≤95% similarity index for defining a genotype. Seven distinct genotypes (defined as AFLP1 to AFLP7) were identified among the 19 *C*. *orthopsilosis* isolates recovered from 16 individual patients in Kuwait with an overall similarity value of 64%. Only two previous studies have studied genotypic heterogeneity among *C*. *orthopsilosis* isolates by AFLP analyses [18,23]. Tavanti et al. [23] first applied AFLP fingerprinting on *C*. *orthopsilosis* strains, collected over an 8-year period and isolated from 11 individual patients and repeat isolates from 2 additional patients, and identified 12 genotypes with an overall similarity index of 73%. Similarly, De Carolis et al. [18] identified 6 distinct genotypes among 8 individual patient isolates and repeat isolates from another patient with a similarity index of 74%. Similar to our study, repeat isolates in these two studies also exhibited nearly identical AFLP patterns with ≥98% similarity index [18,23]. Thus, *C*. *orthopsilosis* isolates from Kuwait exhibited a lower similarity index (64%) than that described in two previous studies. However, a remarkable finding of our study was the occurrence of a dominant genotype (AFLP1) that was shared among 10 individual patient isolates. Interestingly, all the 10 *C*. *orthopsilosis* isolates in AFLP1 also belonged to a single haplotype (ITSB) based on ITS region sequence data even though the isolates were collected over a long period of time (8 years). On the contrary, all 4 and 2 individual patient isolates in ITS region sequence-based haplotypes ITSA and ITSC, respectively, belonged to unique AFLP genotypes. These findings support recent observations that some strains remain in the hospital environment for years forming reservoirs of infection for susceptible patients [6].

Until recently, only two subspecies, exemplified by Type 1 (ITSA haplotype) and Type 2 (ITSB haplotype) have been recognized among clinical *C*. *orthopsilosis* isolates based on ITS region sequencing and the structure and analysis of the mating type locus [13,42,43]. The presence of a third subspecies (ITSC haplotype) has been noted recently among clinical *C*. *orthopsilosis* isolates from Brazil [41] and this haplotype has also been found in our present study. Although *C*. *orthopsilosis* isolates with slightly different ITS region sequences have been described previously (P3 variant and C_ort_T2_90_125 isolates), these differences were minor involving a single nucleotide position [42,43]. On the contrary, *C*. *orthopsilosis* ITSC haplotype isolates show sequence variations at 4 and 8 nucleotide positions with ITSA (Type 1) and ITSB (Type 2) isolates, respectively. Whole genome sequence of the intermediate sequence isolate (C_ort_T2_90_125) described by Sai et al. [43] has been determined and genomic comparisons with Type 1 isolate have shown that C_ort_T2_90_125 isolate represents a hybrid, generated as a consequence of a hybridization event between the two (Type 1 and Type 2) *C*. *orthopsilosis* subspecies [45,46]. Although AFLP genotyping showed that our ITSC haplotype isolates were distinct strains, the possibility that they were also generated as a consequence of a hybridization event between the two *C*. *orthopsilosis* subspecies cannot be excluded due to lower sensitivity of the AFLP genotyping. Whole genome sequencing and genomic comparisons of ITSC isolates will be required to confirm or exclude this possibility.

A limitation of our study is that we have tested only few epidemiologically unrelated strains of *C*. *parapsilosis* sensu stricto, *C*. *orthopsilosis*, *C*. *metapsilosis* and *L*. *elongisporus* to validate the mPCR protocol due to non-availability of such isolates in our culture collection.

## Conclusions

A simple, low-cost mPCR assay has been developed for rapid detection and differentiation of clinical *C*. *parapsilosis*, *C*. *orthopsilosis*, *C*. *metapsilosis* and *L*. *elongisporus* isolates for rapid screening of clinical *C*. *parapsilosis* sensu lato isolates in a clinical microbiology laboratory. The occurrence of *C*. *orthopsilosis* and *C*. *metapsilosis* among clinical isolates can now be easily studied from various countries/geographical locations for epidemiological investigations. We also detected genotypic heterogeneity among clinical *C*. *orthopsilosis* isolates in Kuwait and found a dominant genotype (ITS region haplotype ITSB and AFLP1) that first appeared nearly 8 years ago and has persisted in Kuwait hospitals highlighting the possible existence of reservoirs of infection.

## Supporting Information

S1 Fig Colony characteristics of *C*. *parapsilosis* (A), *C*. *metapsilosis* (B), *C*. *orthopsilosis* (C) and *L*. *elongisporus* (D) on CHROMagar Candida after 3 days of incubation at 30°C. (TIF)

S1 Table Differentiation of *C*. *orthopsilosis* into distinct genotypes based on sequences at specific nucleotide positions in the ITS region of rDNA. (DOCX)
