# Supplementary figures and images for: Simple, Low-Cost Detection of Candida parapsilosis Complex Isolates and Molecular Fingerprinting of Candida orthopsilosis Strains in Kuwait by ITS Region Sequencing and Amplified Fragment Length Polymorphism Analysis
Source: PLoS One. 2015 Nov 18;10(11):e0142880. doi: 10.1371/journal.pone.0142880 (PMC4651534; doi:10.1371/journal.pone.0142880)

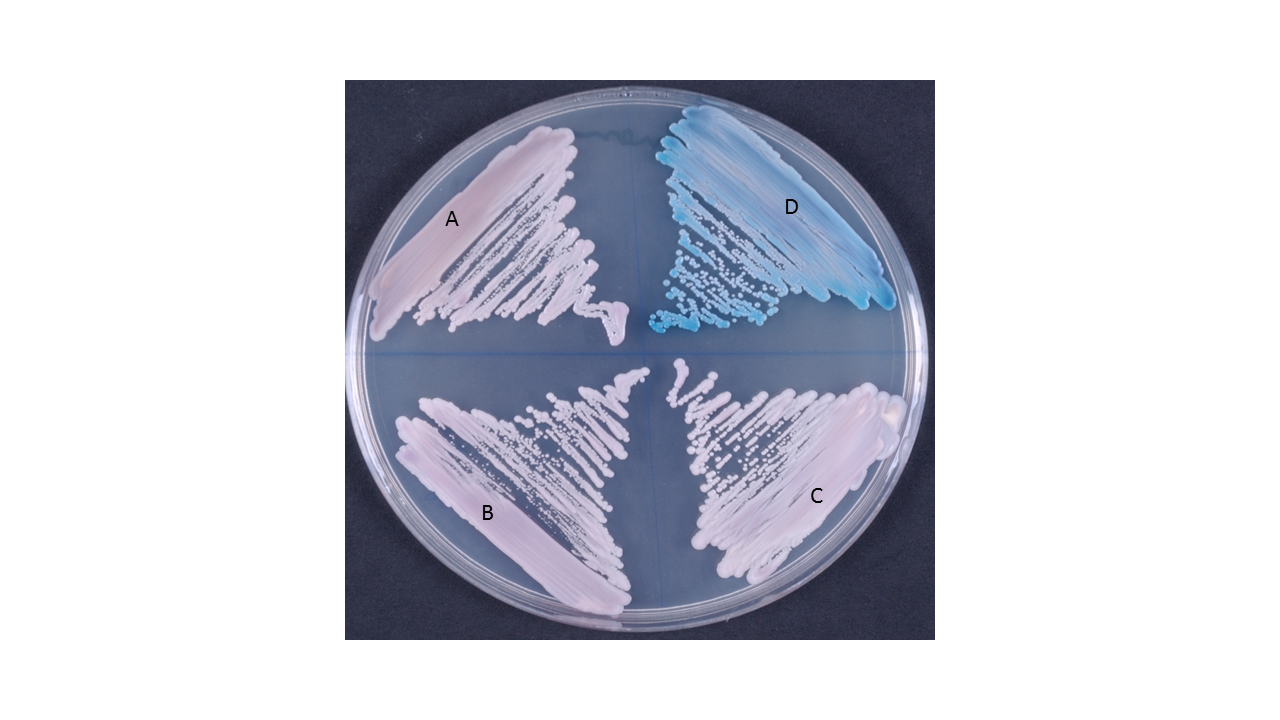

Supplement: S1 Fig — (TIF) [file pone.0142880.s001.tif]
