# Supplementary material for: Simple, Low-Cost Detection of Candida parapsilosis Complex Isolates and Molecular Fingerprinting of Candida orthopsilosis Strains in Kuwait by ITS Region Sequencing and Amplified Fragment Length Polymorphism Analysis
Source: PLoS One. 2015 Nov 18;10(11):e0142880. doi: 10.1371/journal.pone.0142880 (PMC4651534; doi:10.1371/journal.pone.0142880)
Supplement: S1 Table — (DOCX) [file pone.0142880.s002.docx]

**Supplementary Table 1**. Differentiation of *C. orthopsilosis* into distinct genotypes based on sequences at specific nucleotide positions in the ITS region of rDNA

| Haplotype | No. of | Representative | ITS region | Nucleotide at ITS region of rDNA position^b^ | | | | | | | | |
| --- | --- | --- | --- | --- | --- | --- | --- | --- | --- | --- | --- | --- |
|  | isolates | isolate no. | length^a^ (bp) | 58 | 78 | 79 | 109 | 142 | 143 | 144 | 145 | 414 |
| ITS-A | 5 | Kw301-97 | 516 | T | T | T | T | A | T | T | T | T |
| ITS-B | 11 | Kw1782-06 | 519 | Ins T | T | T | T | T | A | Ins T | T | Ins T |
| ITS-C | 3 | Kw96-12 | 515 | T | Del | Del | C | A | T | Ins T | A | T |

^a^The ITS region length included the first nucleotide of primer ITS1 and last nucleotide of primer CTS1R used for the amplification of ITS region of rDNA.

^b^The nucleotide positions are shown relative to *C. orthopsilosis* Kw301-97 (EMBL accession no. FM172981) that belongs to haplotype ITS-A and its sequence is 100% identical to the reference *C. orthopsilosis* strain (ATCC 96139, GenBank accession no. NR_130661)
